# Quantification of cell identity from single-cell gene expression profiles

**DOI:** 10.1186/s13059-015-0580-x

**Published:** 2015-01-22

**Authors:** Idan Efroni, Pui-Leng Ip, Tal Nawy, Alison Mello, Kenneth D Birnbaum

**Affiliations:** Center for Genomics and Systems Biology, New York University, 12 Waverly Place, New York, 10003 USA

## Abstract

The definition of cell identity is a central problem in biology. While single-cell RNA-seq provides a wealth of information regarding cell states, better methods are needed to map their identity, especially during developmental transitions. Here, we use repositories of cell type-specific transcriptomes to quantify identities from single-cell RNA-seq profiles, accurately classifying cells from *Arabidopsis* root tips and human glioblastoma tumors. We apply our approach to single cells captured from regenerating roots following tip excision. Our technique exposes a previously uncharacterized transient collapse of identity distant from the injury site, demonstrating the biological relevance of a quantitative cell identity index.

**Electronic supplementary material:**

The online version of this article (doi:10.1186/s13059-015-0580-x) contains supplementary material, which is available to authorized users.

## Background

Many important events in development and disease involve transitions between different cellular identities, demanding methods that can follow cells as they differentiate, undergo reprogramming to highly potent states, or transdifferentiate during tissue regeneration. The development of single-cell RNA-seq technology [1-4] has provided insights into states of individual cells, permitting the analysis of cellular trajectories during dynamic periods of development. Single cell analyses have enabled cellular states to be examined for rare cells in early development as they undergo differentiation [5,6] and during transitions from stochastic to stereotypical states in cellular reprogramming [7].

In order to identify distinct cell types amongst heterogeneous cell populations, single cell studies have mostly relied on unsupervised clustering techniques [4,6,8]. These techniques utilize RNA-seq profiles of the cells themselves to group the cells based on similarity, after which, in a *post hoc* analysis, known markers are used to map cell identity onto clusters [8].

However, cell type classification is complicated by the fact that extrinsic factors, such as differences in micro-environments or transient physiological responses, can manifest in large expression changes that contribute to variability between cells. Methods that use whole-transcriptome correlation are thus biased by physiological and other batch effects. Classification is further complicated by biological noise, resulting from stochastic, burst-like transcription events [9] and the substantial technical noise inherent in single cell sequencing data [4,10,11]. This technical noise stems from the low number of mRNAs present in single-cell samples and the stochastic nature of the amplification and sample preparation process [11,12]. Thus, indices of cell identity must be robust to biological and technical noise in single cell measurements but also sensitive enough to detect weak signals that represent mixed cell character or transitional states.

Comprehensive repositories of cell and tissue expression profiles are a valuable resource for quantifying both cell identity and transitional or mixed cell states using a supervised approach. Such repositories are available for a growing number of systems, including the mouse brain [13,14], human and mouse hematopoietic system [15-17], various cancer types [18], and the plant root [19,20] and shoot [21].

An important consideration that has not been formally addressed is the selection of genes that can serve as cell identity markers for single cell experiments. Tissue and cell type-specific reference libraries are typically dominated by noisy biological patterns with respect to cell identity [22], where most markers are expressed in multiple cell types, even if they have relatively restricted expression domains or temporal patterns. Extreme filtering of large datasets for highly specific markers reduces the power to detect cell identity in noisy systems, as small numbers of markers make inferences susceptible to noise. Using a large number of markers requires the incorporation of less specific markers, decreasing the specificity of the identity call. Thus, there is an optimal number of markers for detecting identity, which may vary between experimental systems.

To address these issues, we propose an approach for cell type classification that utilizes sets of informative markers, which are not required to be uniquely expressed in a single cell type. To select appropriate markers, we adapted an information-theory based approach that analyzes technical and biological variability in expression across environments and expression domains [22] and utilizes this information to generate an index of cell identity (ICI) for single-cell mRNA-seq samples. The ICI of a given cell represents the relative contribution of each identity as evaluated from a reference dataset of cell profiles. The use of a quantitative score allows the identification of transitional and chimeric identities. We apply our method to single cells extracted from the *Arabidopsis* root meristem, which has a wealth of cell type- and developmental stage-specific expression profiles [19,20,23] and to a population of 365 single cells previously isolated from five human glioblastoma tumors [24]. We show that our method is accurate in classifying single cells, can optimize marker selection, and performs well with plant and animal datasets.

**Figure 1:**
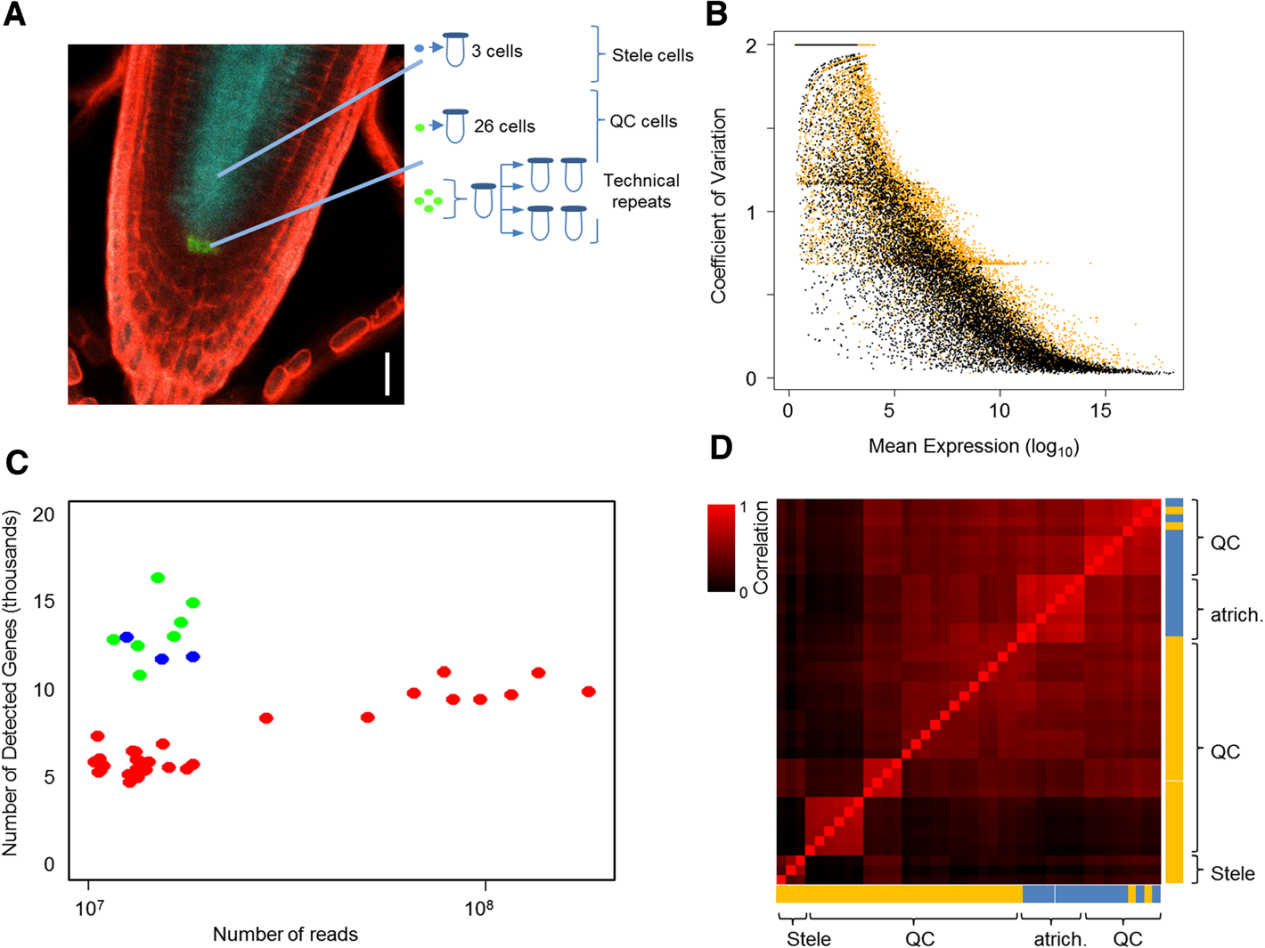
**Characterization of single-cell profiles from the** ***Arabidopsis*** **root. (A)** Confocal image showing the markers used and cell types collected in this study. *WOX5:GFP* marks the QC, and *WOL:CRE-GR 35S:lox-CFP* is a lineage marker for the stele. **(B)** Noise (coefficient of variation) in the technical repeats (black) and in four individual QC cells from the same batch (orange). **(C)** Number of genes detected as a function of sequencing depth for different cell types. Red, QC; blue, stele; green, atrichoblasts. **(D)** Hierarchical clustering of a cross-correlation matrix between the single cell profiles. Batches are color coded (blue [10]; orange, this study) to show batch effects.

To assess the utility of our method in classifying transitional and complex identities, we use it to analyze plant cells isolated from regenerating roots, as plant cells are known to have high levels of developmental plasticity. Roots grow through rapid cell division in growth zones called meristems that contain a stem cell niche. At the center of the stem cell niche is a group of cells with low-mitotic activity known as the quiescent center (QC) [25] (Figure 1A). When the root tip, including the stem cell niche, is completely removed, missing cell types rapidly regenerate from differentiated cells in the remaining stump within 24 to 48 hours [26,27]. As a test of our methods to quantify cell identity, we followed a set of regenerating cells by permanently marking the root stele tissue, an internal tissue that contains early differentiation stages of vascular cell types, and isolating individual, marked cells immediately following tip excision and at 16 hours after excision. Our method detected a transient loss of original identity in cells throughout the stele during the regeneration process, which is validated by fluorescent marker analysis. These results suggest that regeneration involves a rapid, transient, and widespread loss of tissue identity during the reorganization of the root tip.

## Results

### Generation of single cell profiles of the root meristem

To generate single-cell mRNA-seq profiles from different tissues in the well-characterized *Arabidopsis* root, isolated *Arabidopsis* root tips with fluorescently marked tissues were treated with cell-wall digesting enzymes for approximately one hour, and individual cells were collected using a glass mouth pipette (see Materials and methods). This procedure allowed us to visually confirm the presence of a single fluorescent cell in the capillary pipette. Overall, 31 individual cells were collected and processed: 24 QC cells (marked by *WUSCHEL-Related Homeobox 5* (*WOX5:GFP*) [28] and collected in four batches), and 7 stele cells (clonally marked with *WOODEN LEG (WOL) WOL:CRE-GR 35S:lox-CFP*; Figure 1A) that consisted of three cells collected immediately after removing the stem cell niche (0 h post-excision) and four cells collected at 16 h post-tip excision.

To assay technical noise, technical replicates were generated by pooling four of the QC cells, lysing and splitting the mix into four aliquots (Figure 1A). We preferred the use of a small number of cells rather than a diluted sample of many cells, to better represent technical variability due to working with small quantities of RNA at all steps. Each sample was processed and amplified individually according to previous protocols [1], and amplified cDNA was sequenced using the Illumina platform to generate 50 bp single-end reads. Overall, 80 to 87% of the reads in each sample mapped to the *Arabidopsis* TAIR10 genome and over 95% of those reads aligned to annotated genes. We did not detect any transcripts from intergenic regions since only a few reads aligned to non-annotated regions (<0.01% of all mapped reads), and none of the intergenic read clusters spanned a region greater than 300 bp (data not shown).

Consistent with earlier single-cell RNA-seq studies [10,11], technical variation was highly correlated with expression level (Figure 1B). This technical noise was greatest at low expression levels; however, at high expression levels, variation between individual QC cells is greater than the variation between the pooled-and-split cells (*P* < 0.05, Kolmogorov-Smirnov test; Figure 1B), suggesting that there is biological variability between QC cells that exceeds the technical variability.

Since we sought to assess cell identity, we first examined the effects of read depth and the transcriptional patterns of known markers in the well-characterized root system. For all subsequent analyses, we combined our single-cell data with 13 previously collected root cells [10]: 6 QC cells (from the same reporter line used in our experiments), and 7 epidermal atrichoblast cells, marked by the reporter *GLABRA2*(*GL2*)*:GFP*. Together, this allowed us to assess well-documented markers in three different cell types and also examine batch effects stemming from sampling the same set of cells in different labs.

The number of genes detected in each sample was similar among the stele cells (approximately 14,000 genes/cell) but varied between epidermal cells (11,000 to 18,000 genes/cell). We sequenced some of our QC cells to higher depth to assess the effect of total reads per cell on the number of detectable genes. The analysis showed that the libraries saturate at 5 × 10^7^ reads/cell. We detected 4,312 more genes on average than cells sequenced at a depth of less than 5 × 10^7^ reads/cell (Figure 1C). This suggests that many more genes are expressed in QC cells than previously detected by shallow sequencing [10]. Furthermore, we detected a large number of transcripts expressed at a low level that show widespread, non-specific transcription, possibly arising from sporadic transcriptional events.

We found several examples of low level marker expression in ‘unexpected’ cell types, including the QC marker *WOX5* in one stele cell, the stele-specific gene *SHORT ROOT* (*SHR*) in four QC cells, epidermal marker expression in one QC cell, and the endodermal/QC-specific gene SCR in six of the seven atrichoblast samples (Figure S1A in Additional file 1). To rule out the possibility that the detection of low-level transcripts arises from ruptured root cells or other contamination during cell isolation, we examined the expression of pollen-specific genes, a set containing many transcripts that are unique to the specialized gamete and are not detected above noise in roots of pooled cells [29]. Indeed, sporadic, low-level expression of many pollen-specific genes was detected (Figure S1B in Additional file 1), further supporting the observation that the sampled cells express non-specific transcripts at low levels.

QC cells are located together in close proximity in the root, are morphologically indistinguishable, rarely divide, and are considered to be homogenous [25]. The depth of coverage for these cells allowed us to assess the consistency in detecting transcripts across theoretically identical cells. This analysis showed that even single-cell profiles from the homogenous QC show wide variation in their transcriptional composition. The cumulative number of detected genes in all samples declines at a nearly constant rate with each new QC sample regardless of which lab conducted the experiment (Figure S1C in Additional file 1). Thus, while we detected about 10,000 genes in any given QC cell, only 943 genes were detected in all 30 QC cells. A total of 15,492 genes were expressed in at least one QC cell. The number of common highly expressed genes diminishes at a higher rate, with only 11 genes common to all 30 QC cells (Figure S1C in Additional file 1). These phenomena appear common in single-cell profiles, as we and others have observed similar trends in both plant [10] and recent mouse studies using an *in vitro* transcription amplification technique [11] (Figure S1D in Additional file 1). Our analysis highlights two important features of single-cell profiles that should be considered when assessing identity from known markers. First, single-cell profiles are likely to display low-level sporadic expression of transcripts from ‘ectopic’ identities, and second, the absence or low-expression of any given cell type-specific marker in these profiles is highly likely.

### A method for classification of cell identity

The biological and technical noise associated with the root single-cell profiles suggests that robust classification of cell identity is not a trivial problem. Indeed, in a hierarchical clustering of pair-wise cell-cell Pearson correlation values (Figure 1D), cell profiles grouped mainly according to lab of origin rather than tissue of origin. This outcome makes cell affinity comparisons across labs and experiments problematic.

To establish a method for robust quantification of cell identity, we utilized an extensive reference set of tissue-specific expression profiles collected over a decade of research using cell sorting and profiling with the ATH1 Affymetrix microarray [19,20,23,30-32]. To identify markers which can be used to quantify identity in single cells, we adapted an information theory-based method to calculate Spec values, which represent the amount of information each transcript provides for determining cell identity [22]. From the cell type-specific dataset, we selected microarray profiles mostly from non-overlapping root tissues, collected under a variety of physiological states. These profiles combined publicly available data and new microarray profiles of the QC that were generated for this study (Additional file 2). The use of multiple physiological states to represent single identities ensured a set of robust markers for a given cell identity.

**Figure 2:**
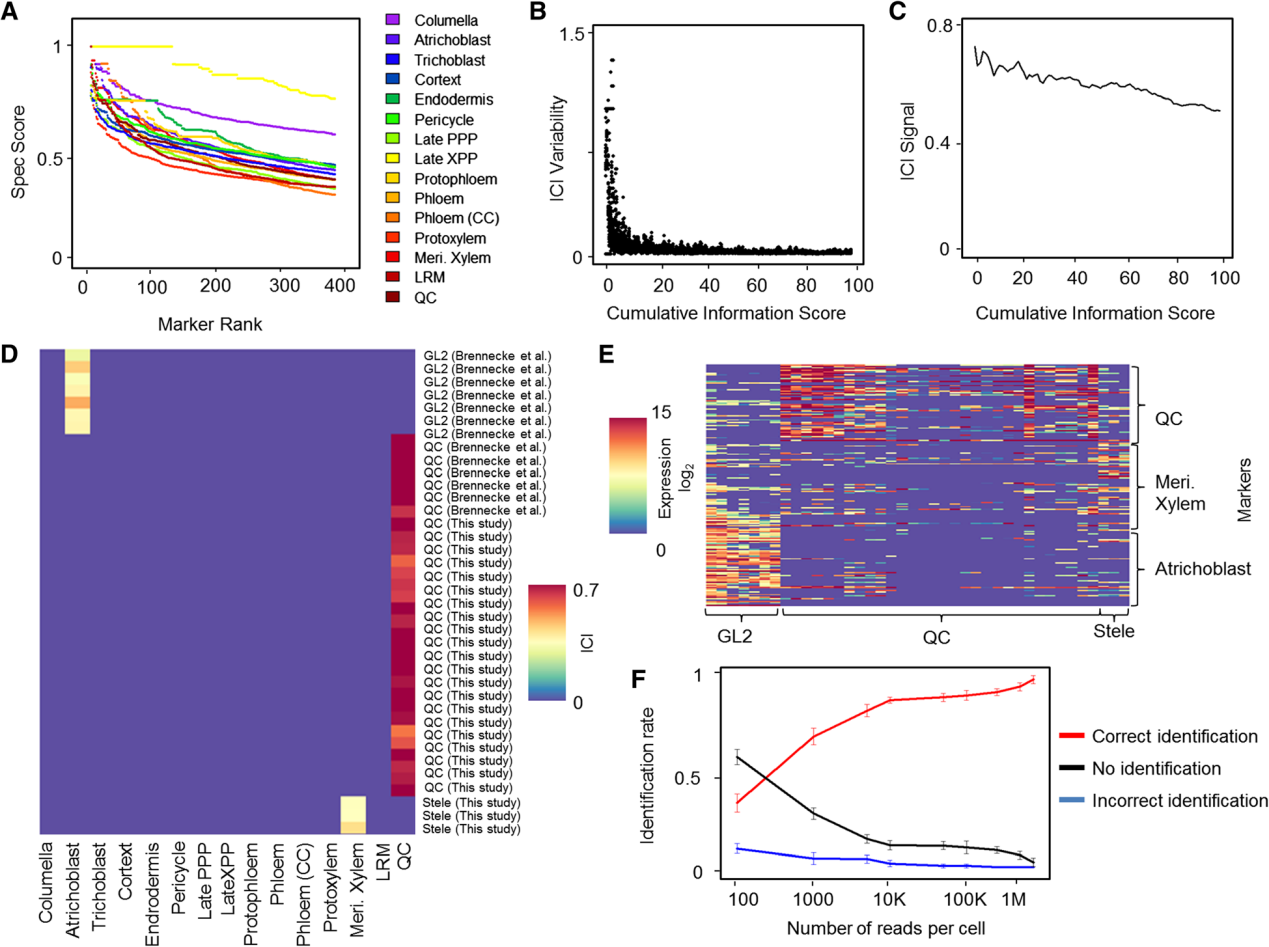
**Information-based index of cell identity robustly assigns cell type to single cells. (A)** Spec score with increasing marker set sizes in different root tissues measuring level of transcript uniqueness in each cell type. CC, Companion Cell; LRM, Lateral Root Meristem; PPP, Phloem-Pole Pericycle; XPP, Xylem-Pole Pericycle. **(B)** ICI variability as a function of the number of markers used. Variability is measured as the Euclidean distance between ICI vectors for cumulative information threshold *i* and *i* + 1*.* **(C)** ICI signal for the 30 plant cells used in the study as a function of the number of markers. **(D)** ICI output for the single cells used in this study, showing the predicted identity in the heatmap and the cell type marked by the reporter used to collect the cells. **(E)** Expression of selected markers for atrichoblast, stele, and QC in the single cells. Note the overall tendency of marker specificity and substantial noise from either technical or biological sources. **(F)** Proportion of cell identities called correct (red), unidentifiable (black), or incorrect (blue) at varying sequencing depths.

Calculating Spec values requires binning of the data, and we sought to optimize bin selection to obtain robust markers. We assigned transcript levels into one of two bins (high/low expression), separating background and true expression (Materials and methods). We set the cutoff for background expression under the assumption that optimal markers would be expressed at background levels in the majority of cells and true expression would be relatively rare. This routine established a range for the background cutoff, within which we established a precise cutoff by finding the cutoff value that maximized the Spec score (Materials and methods). Using this routine, the algorithm automatically selected markers for which high expression in specific cell types was well separated from the background levels in the majority of cells. Markers were ordered according to their Spec scores, and we determined markers for each of the 15 root tissues by selecting genes with the highest Spec score for a given tissue (Figure 2A; Additional file 3). Thus, Spec scores provided the ability to rank markers according to their information content on cell identity.

For each tissue, a marker set was chosen to provide the same level of information. Some tissues have more highly informative markers than others (Figure 2A). Therefore, Spec information scores were added until they reached a defined constant, resulting in more distinct tissues having fewer markers than less distinct tissues. In this way, the most highly informative markers are used to diagnose each cell identity and each cell type is given the same level of diagnostic power. Based on these markers, we compute an ICI for each tissue as follows. Using RNA-seq read counts from each single-cell profile, we calculate the mean expression of all genes in the predefined marker set and weight each gene by its Spec score for the particular identity. To down-weight cases of sporadic noisy expression of individual markers and control for false positives, we then adjust the score by the proportion of markers expressed (expression level >0), effectively weighting in favor of identities for which many markers are detectable. For any given single cell that is queried, the procedure then determines an ICI for each cell type that is then normalized to a range of 0 to 1. This method generates a relatively simple index that is robust to low-level sporadic expression (false positives) and frequent absence of a given marker (false negatives). To generate a confidence measure, we randomized marker sets of equal size to the original and performed 1,000 permutations to establish a null distribution of ICIs for each cell type (see Materials and methods). We used a *P* < 0.05 cutoff to determine significant ICI above background.

The above procedure weights the contribution of a marker to the ICI by its pre-determined specificity in predicting a cell type. However, one free parameter in our method is the cumulative information threshold for marker selection, which determines the number of markers used per tissue. We analyzed the effect of this parameter on detection power by varying marker set size. We calculated the ICI for each cell from both our own experiments and the previously generated QC and epidermal cell profiles. Within a wide range of cumulative information threshold, representing relatively small or large marker sets, the procedure was able to classify all cells correctly (Figure S2A in Additional file 1). However, using a very low cumulative information threshold yielded results that were highly sensitive to the threshold, as indicated by a local variability measure (hereafter, ‘ICI variability’; Figure 2B). On the other hand, increasing the cumulative marker threshold resulted in lower mean maximal ICI (hereafter, ‘ICI signal’; Figure 2C; Figure S2 in Additional file 1) because a greater number of less-specific, ‘noisy’ markers were included in the analysis. The result illustrates a tradeoff between robustness (ICI variability) and signal strength (ICI signal) in determining marker set size, where ranking markers for informativeness permits an optimization between the two effects. The analysis suggests that the cumulative information threshold should be set in a range where ICI variability is stable and minimal, while ICI signal is high. As marker behavior and specificity vary between experimental systems [22], the optimal threshold needs to be empirically determined. To maximize robustness and signal strength in the plant dataset, we selected a cumulative information threshold value of 20, which was used for the rest of the study (Additional file 3).

Using this procedure, all cells picked using the highly specific QC marker *WOX5:GFP* were identified as QC regardless of the lab that generated them, showing the method is robust to batch effects (Figure 2D). The three stele cells scored the highest for meristematic xylem tissue. This tissue represents early developmental stages of the xylem and is contained within the tissues marked by the *WOL* promoter [33]. With respect to epidermal cells, the previous single-cell study that collected atrichoblast (non-hair epidermal cells) using *GL2:GFP* could not distinguish the isolated cells from a closely related subtype (trichoblast or hair cells), using transcriptome similarity-based methods [10]. However, our information-based approach correctly identified the cells as atrichoblast and not trichoblasts, showing the method can sensitively discriminate very subtle differences in cell subtypes (Figure 2D) and is robust to the noise of single-cell profiles (Figure 2E). We investigated the ability of the ICI to differentiate cell types and account for noise by analyzing multidimensional scaling plots using the Pearson correlation as a distance measure. When using the expression of the markers identified by our algorithm (information threshold of 20,258 markers), cells could be separated by tissue, but noise and batch effects were still very apparent. This noise was evidenced by QC cells occupying a large space, making it difficult to assign them to a coherent group (Figure S3A in Additional file 1). In contrast, using the ICI vectors, identities were clearly distinct, while batch and lab-dependent effects were no longer evident (Figure S3B in Additional file 1).

### Sensitivity to read depth and chimeric states

Single-cell studies are now being generated for hundreds and thousands of cells [8], which means each cell is sequenced at a very shallow depth. To assay the sensitivity of our method at different read depths, we generated shallow read depth samples by resampling and determined the rates of correct calls for the 40 single root cells. Our method was sensitive enough to classify the correct tissue among the 15 possible cell types at high confidence 87% of the time with 10,000 reads/cell and 91.3% of the time with 500,000 reads/cell (Figure 2F). Importantly, there were very few incorrect classifications even at very shallow read depths (1.75% and <0.5% at 10,000 and 50,000+ reads/cell, respectively; Figure 2F). These results demonstrate the feasibility of our method in highly multiplexed experiments with shallow sequencing depths.

In addition, one promising application for single-cell transcriptomics is the ability to track cells as they undergo differentiation or cellular reprogramming and to identify chimeric, transitional states or prior state memory. To test our algorithm on mixed cell states, we generated mixed cell profiles by randomly sampling reads from either QC and atrichoblast cells or QC and meristematic xylem cells. Using a stringent threshold, our algorithm identified both cell types in a simulated single cell in 62% and 21% of the cases in QC-atrichoblast and QC-meristematic xylem mixed cells, respectively (Figure S4 in Additional file 1). As chimeric identity dilutes both identities, the lack of any significant identity justifies a lower threshold to explore potential mixed cell states. Indeed, lowering the threshold resulted in detection of multiple identities in 75% and 57% of the simulated QC-atrichoblast and QC-meristematic xylem mixed cells, respectively (Figure S4 in Additional file 1). The rate of false positives at a low threshold was small, as non-pooled cell identities were detected at a low proportion of 2 to 3% (Figure S4 in Additional file 1).

### Classification of single cells from human glioblastoma tumors

**Figure 3:**
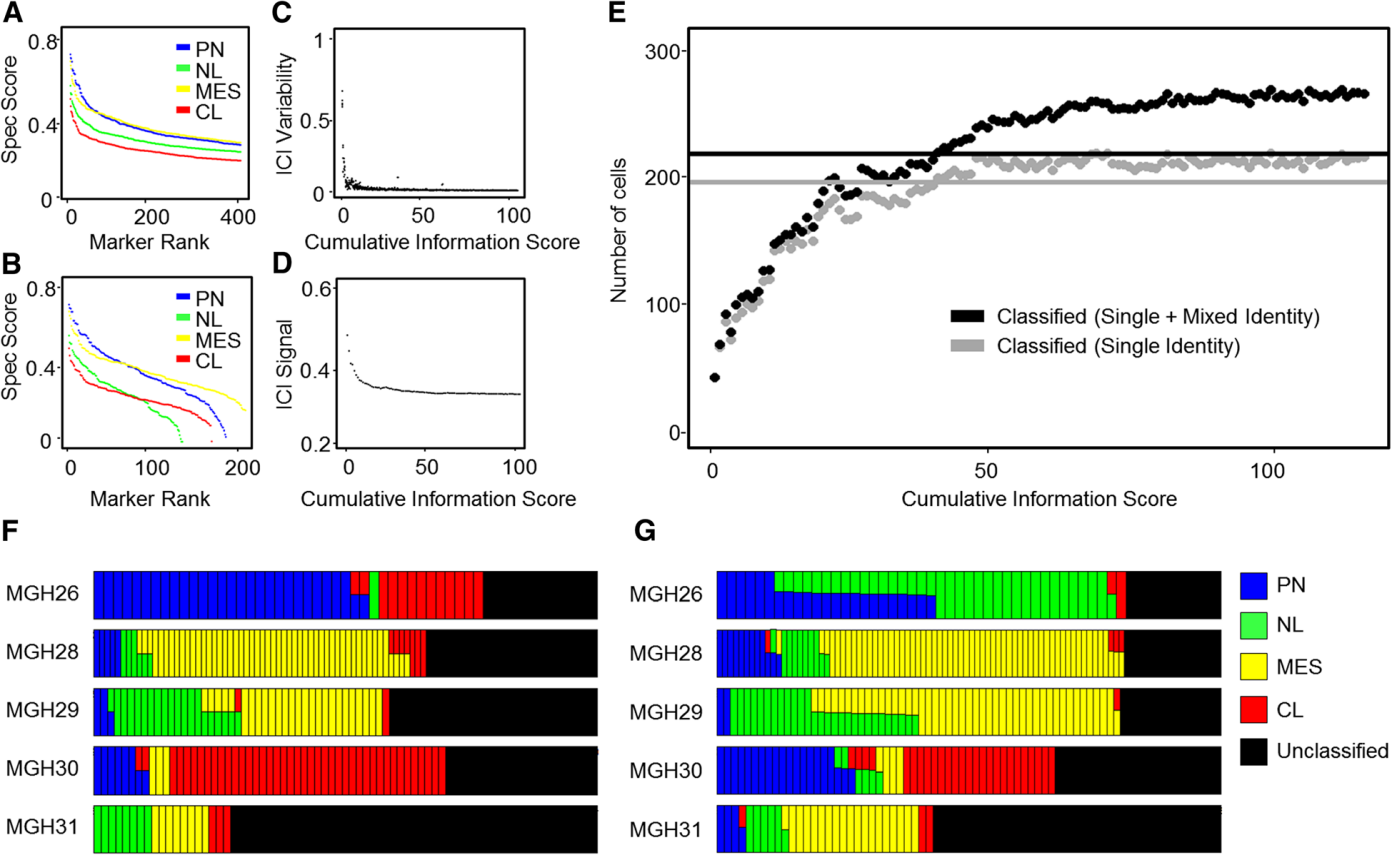
**Marker analysis and identity scores of cells from glioblastoma tumors. (A,B)** Spec scores for the glioblastoma markers identified by our algorithm **(A)** and for the TCGA markers previously published [34] ordered by rank. Tumor subtypes are: CL, classical; MES, mesenchymal; NL, neural; PN, proneural. **(C,D)** ICI variability **(C)** and ICI signal **(D)** for the glioblastoma cells*.* **(E)** Number of classified cells in all five tumors as a function of the marker information threshold. Black indicates all classified cells including mixed identities. Gray indicates cells classified as a single identity. Lines indicate number of cells classified in a previously applied algorithm [24]. **(F,G)** Identity calls for individual cells from each subtype in each of the five tumors, using the algorithm and markers in a previous analysis [24] **(F)**, and using the ICI method with a cumulative information threshold of 110 **(G)**. Each column represents a single cell, and the color-coded bars represent relative identities.

To test our method in a different system, we utilized a previously profiled collection of 365 cells from five human glioblastoma tumors, sequenced at low depth [24]. Using The Cancer Genome Atlas (TCGA) whole tumor transcriptome data from four subtypes of glioblastoma [34], we identified high information markers among these tumor types. Overall, glioblastoma subtype markers had significantly lower Spec scores than root tissues (Figure 3A; Additional file 4), indicating a significant overlap in transcript expression between the different subtypes. Previous analysis by TCGA identified a set of 840 marker genes that can differentiate between the subtypes [34]. However, our data show that while part of the TCGA marker set has relatively high Spec scores, a substantial part of the set has low scores (Figure 3B), suggesting that a more optimal subset could be identified. In comparison with the plant dataset (Figure 2B,C), the ICI variability decreased more rapidly for the glioblastoma dataset while ICI signal remained stable with increasing marker set size (Figure 3C,D), indicating a wide possible range for an optimal marker number.

A previous attempt to classify the cells using the mean expression of the TCGA markers could identify 59% of the cells [24] (Figure 3E). To identify an optimal marker number, we measured the number of classified cells while varying cumulative information thresholds. Increasing the cumulative information threshold consistently improved the number of identified cells of both single and mixed fates, and could surpass the performance of previous methods [24] (Figure 3E). Using a cumulative information threshold of 110 (1,368 marker genes; Additional file 4), our method was able to classify 72% of these cells (Figure 3E).

Both methods show that all tumors were of mixed identities with cells representing different subtypes at various proportions [24] (Figure 3F,G). The proportions of the subtypes in the tumors predicted by our method were similar to those previously verified [24], with the exception of the identification of a previously uncharacterized neural population in tumor MGH26 (Figure 3F,G).

Overall, this analysis shows that the ICI method is broadly applicable and can provide formal methods to optimize marker set selection for the diagnosis of cell identity. In particular, ranking markers based on Spec values allows the use of many informative markers, optimizing use of previously published marker sets [34].

### Classifying transitional states during root regeneration

**Figure 4:**
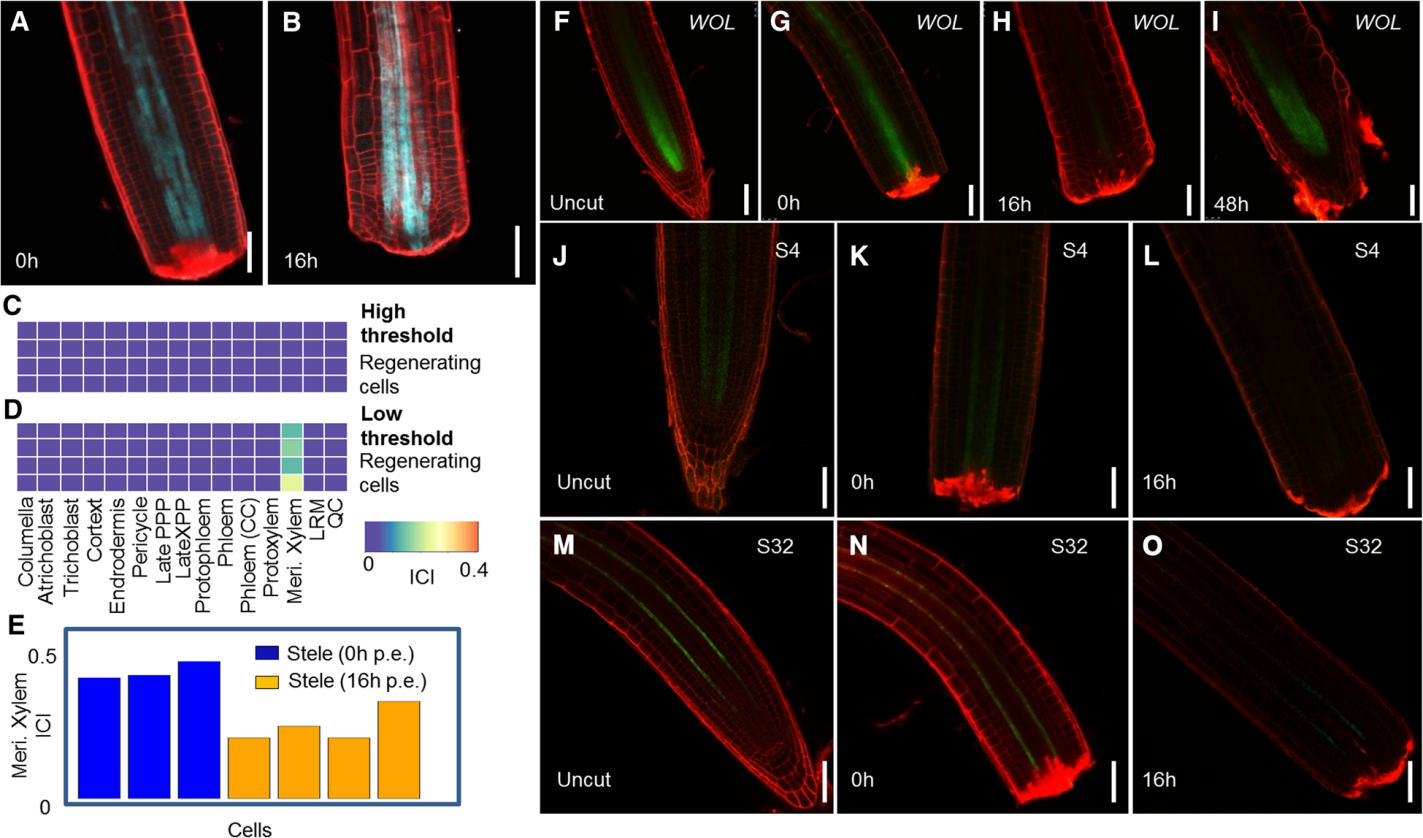
**Quantitative loss of identity in regenerating root vascular cells. (A,B)** Induced *WOL:CRE-GR 35S:lox-CFP* roots, immediately after **(A)** and 16 hours **(B)** following root tip excision. **(C,D)** Significant ICIs (*P* < 0.05 **(C)** and *P* < 0.2 **(D)**) for the four cells isolated from the regenerating root shown in **(B)**. CC, Companion Cell; LRM, Lateral Root Meristem; PPP, Phloem Pole Pericycle; XPP, Xylem Pole Pericycle. **(E)** Meristematic xylem ICIs for stele cells. **(F-O)** Confocal images of *WOL:GFP* **(F-I)**, S4 **(J-L)** and S32 **(M-O)** plants before **(F,J,M)**, immediately after **(G,K,N)**, and at 16 **(H,L,O)** and 48 **(I)** hours post-tip excision. Note that expression of vascular markers is greatly diminished at 16 hours.

In order to test the algorithm on cells that undergo regeneration, we analyzed the cells from the root stele tissue 16 hours after excision of the root tip (Figure 4B) and removal of the stem cell niche [27]. After decapitation, the root meristem reorganizes, reforming the missing stem cell niche and columella from the remnant stump in the position of the former stele [27]. We engineered a stele-specific marker, *WOL:CRE-GR 35S:lox-CFP* (Figures 1A and 4A,B; Materials and methods) for this experiment The use of this ‘memory’ marker allowed us to definitively assign the cell type of origin of the marked cells during tissue regeneration [27].

At a high significance threshold, stele cells from the regenerating roots had no significant identity (among the 15 cell types queried; Figure 4C), indicating a quantifiable loss or decrease in cell identity. When the significance threshold was relaxed, meristematic xylem was still the highest identity in these cells (Figure 4D), although the ICIs were significantly reduced (Student’s *t*-test, *P* < 0.05; Figure 4E). Indeed, expression of most meristematic xylem markers was reduced, although still present (Figure S5A in Additional file 1). In addition, expression of individual known stele and vascular differentiation markers, such as the stele-specific vascular developmental regulator *WOL*, was reduced in the regenerating cells (Figure S5B in Additional file 1). The analysis suggests internal root tissue undergoes a stage of identity loss during the regeneration process. As cells are collected following disassociation, their exact spatial location cannot be confirmed. However, we inferred from these single-cell profiles and previous work that the cells selected were not in the distal end of the cut root stump, as we have shown that these cells near the injury site undergo rapid reprogramming [27]. Thus, the result leads us to hypothesize that tissues relatively distant to the cut site undergo an attenuation of identity, even though they are possibly outside of the region where cell identities rapidly reprogram.

To corroborate these results, we examined the expression of a labile *WOL:GFP* marker during the regeneration process. In agreement with our single-cell data, expression of *WOL* is transiently down-regulated throughout the regenerating stele at 16 hours and recovers following the regeneration of the root at 48 hours following excision (Figure 4F-I). We also examined the markers S4 and S32, which mark the internal meristematic xylem and protophloem tissues, respectively [30]. Both markers were down-regulated throughout the meristem by 16 h post-excision (Figure 4J-O). The signal for both markers was still visible at low levels, indicating partial conservation of identity.

The role of this transient loss of internal tissue identity requires further investigation. However, the example shows that we can detect quantitative transitions in cell fate in regenerating tissue, with cells showing signs of a collapse of original cell identity during regeneration. Overall, our techniques provide an approach to quantify cell identity in single cells in a robust and sensitive manner that should be useful in regeneration and reprogramming studies.

## Discussion

Single-cell RNA-seq can be used to gain insights into cellular trajectories and intermediate differentiation steps that are otherwise obscured by bulk analyses. However, classification of cell state is a complex problem due to biological variability among cells and technical noise generated by small-scale amplification techniques. Furthermore, biological replicates are not possible, and methods are still needed to assess the certainty of classification. Here we introduce a formal approach to identify the state of a cell and generate a measure of confidence in the classification of a single or mixed cell identity. Importantly, each cell is diagnosed independently of others based on a stable reference set, so the classification is not fit to a particular experiment and does not change as more cells are sampled. In addition, our method is robust to batch effects that make separate experiments otherwise difficult to compare.

The composition of the marker set used for cell type classification is an important parameter that has not been formally addressed. The biological and technical noise associated with single cell experiments means that the presence or absence of a marker is a probabilistic event. An intuitive solution to the problem is the use of multiple markers for each cell identity to ‘vote’ on classification, as we formalize here in a straightforward index. However, while many markers can be used, not all markers are equally specific, implying tradeoffs when setting the composition of the marker set. For example, intuitively, the presence of several highly specific markers provides a clear diagnosis. However, such an approach would frequently fail in single cell experiments because, as we showed by using several datasets from different labs, including our own, any given marker may be absent in a cell or possibly expressed in an ‘ectopic’ cell. Such anomalies, possibly stemming from sporadic transcription events, are averaged out in pooled cells and thus escape detection. On the other hand, increasing robustness by including more markers diminishes power since markers themselves vary in their informativeness of cell identity. We could observe these tradeoffs in our dataset using a gold standard set of handpicked cells from well-characterized cell types. When using a low number of markers (at a low cumulative information threshold), we observed highly variable ICIs and low performance in calling cell identity. In contrast, using high cumulative information decreased the difference in ICI over background noise and similarly decreased performance.

We can address this tradeoff systematically using our approach, which quantifies the predictive value (information) of markers on cell identity. We determine cell identity using a scoring index that is weighted by the information value of the marker. This approach provides robustness by enabling the use of large marker sets while it minimizes the loss of power by down-weighting less informative markers. In addition, the cutoff for cumulative information can be optimized using ICI variability and ICI signal over increasing marker set sizes. Such analysis does not require a gold standard dataset and can be optimized using experimental data. Overall, this process provides a systematic approach to using a large marker set to increase certainty in cell identity calls without loss of power to detect weak signals. We note that the current limited availability of high-quality single-cell datasets precludes the testing of our method in the full set of scenarios to which it can be applied, and we suggest that future assessment of the method will further clarify its performance.

One application of these methods is determining cell identity during dynamic developmental transitions. Key questions include: How do cells transition from one cell type to another in order to reform injured tissue, and, what is the relative role of dedifferentiation and transdifferentiation in this phenomena [35-37]?

Single cell transcriptomics combined with identity analysis tools can address these questions. The challenge in this problem is confidently distinguishing cell fate transition from natural variability and noise. Unsupervised techniques are not generally designed for discrete or mixed cell classification, as we show that batch effects and other sources of variability do not lead to clear boundaries between clusters of different cell identities. In addition, mixed cell fates would not be expected to necessarily occupy the intermediate space between known cell types. The approach we developed can be used to quantify the loss of identity and to detect the presence of multiple identities in a single cell profile.

In single cells obtained from meristematic vascular tissue of regenerating roots, we were able to detect a previously uncharacterized transient state of identity loss during the regeneration process. These same cells showed a transient loss of expression of *WOL*, a regulator of the plant response to the phytohormone cytokinin [33]. The loss of identity detected was in both meristematic xylem and phloem tissues, and analysis of fluorescent markers corroborated a meristem-wide transient loss of identity in the vascular tissue. This loss of identity may be required to re-establish the differentiation gradient that is normally present in the root meristem and is disrupted by the decapitation of the tip. Although the nature of this transient state requires further investigation, the result illustrates how the ability to sensitively map and quantify changes in cell identity permits high resolution analysis of tissue dynamics during developmental processes. Overall, the ability of our method to quantify multiple identities in single cells makes it a useful tool for future studies in multiple experimental systems.

## Conclusions

We present an information-based method for quantification of cell identities using available cell-type repositories and single-cell RNA-seq profiles. Using our method we identified high-information markers for the *Arabidopsis* root and for human glioblastoma subtypes and used them to classify single cells isolated from theses tissues with high fidelity. Using the method, we identified intermediate states occurring during root regeneration.

## Material and methods

### Generation of marker lines

An inducible, stele-specific lineage marking line (*pH7WOL:CRE-GR*) was generated using a modified CRE:LOX system, where CRE recombinase driven by the well-characterized stele promoter (*WOL*) is sequestered to the cytoplasm by fusion to the glucocorticoid receptor (GR). Upon dexamethasone (DEX) induction, the recombinase excises a terminator flanked by LOX sites to allow the constitutively active plant promoter CaMV 35S to drive fluorescent reporter (ER-*CFP*), permanently marking the induced cell and its daughters. Constructs were introduced into Col-0 backgrounds. The marker lines S4 and S32 were described previously [30].

### Microscopy

For confocal imaging, plants were briefly stained in propidium iodide (PI), mounted in water, and imaged using a Leica SPE confocal microscope with × 20 and × 63 magnification and 405 nm, 488 nm, and 561 nm lasers to excite cyan fluorescent protein (CFP), green fluorescent protein (GFP), and PI, respectively. Voltage and gain settings were held constant for each marker during the time series.

### Plant growth, isolation of single cells and RNA-Seq

Seeds were placed on agar plates (1 × Murashige and Skoog salts (Sigma M5524), 0.5% sucrose ), stratified at 4°C for 48 h, and placed vertically in growth chambers set to 23°C and a 16 h light/8 h dark cycle. *WOX5:GFP* plants were harvested 5 days after stratification, before *WOX5:GFP* signal is apparent in emerging lateral roots. *WOL:CRE-GR 35S:lox-CFP* plants were transferred at 6 days after stratification to induction plates (1 × MS, 0.5% sucrose, 10 μM DEX) for 24 h and then returned to 1 × MS plates. Root tips were then cut as described previously [27] at a distance of 130 μm above the columella, removing the QC and surrounding stem cells, and returned to 1 × MS plates for 16 h. Control plants were left on 1 × MS plates and cut at 130 μm just before harvesting. To isolate the root tips, roots, all grown on a single plate, were subjected to a 10 to 15 minute treatment in cell wall digestion solution, as previously described [38], to dissociate the root tips containing the meristem. Those tips were collected and subject to further treatment for a total of approximately 1 hour. Fluorescently marked cells were isolated under a fluorescence dissecting scope and collected with a glass mouth pipette. To verify single cells were picked, marked cells were transferred to a clean dish, re-pipetted into phosphate-buffered saline, and transferred into an approximate volume of 1 μl of lysis solution. cDNA synthesis and amplification were then preformed as described by [1]. Amplified cDNA was sheared with microTUBs using a Covaris^TM^ S2 System, and the library was prepared using TruSeq DNA Sample Preparation Kit (low throughput protocol) and sequenced on an Illumina HiSeq 2000, generating 50 bp single end reads. Resulting reads were aligned to the *Arabidopsis* genome (TAIR10) using Bowtie2 (parameters: --local -k 6). Gene expression values were calculated by summing the number of reads aligning to any annotated gene exon, using ngsutils. Reads aligning to more than one genomic location were counted as partial read. Genes with coverage of less than 200 bp were removed from downstream analysis. Number of reads per gene was normalized to library depth and log_2_ transformed. For Cel-Seq cells, 40 single QC cells were isolated from *WOX5:GFP* plants and processed according to [3]; data were deposited in Gene Expression Omnibus. Analysis was performed using R 2.15.2.

### Hierarchical clustering and multidimensional scaling

Hierarchical clustering was performed using Pearson correlation scores for log_2_ transformed normalized read counts. Multidimensional scaling was performed using the distance measure (1 - R, where R is the Pearson correlation score between two samples).

### Spec value calculation

Spec score for each marker and tissue were identified according to the algorithm described in [22], using two expression classes and the MAS5 processed dataset of microarray experiments detailed in Additional file 2. The Spec algorithm allows the identification of absence markers in which the given tissue is characterized by lack of expression, designated using negative Spec value. As these markers are not used by our algorithm, negative Spec scores were set to 0. For the plant dataset, we filtered genes shown to be induced by the cell wall digestion process [19] or constitutively highly expressed (median >250). Calculating Spec scores requires binning of the data, but does not prescribe an optimal method to determine bin size [22]. To identify optimal bin sizes, we sought to find the cutoff between background noise and true expression. Since the data were too scarce to infer a distribution and an inflection point, we used an empirical method in which, for each gene, we binned its expression across the samples into *l* equal sized bins, and designated the number of samples in each bin as (*o*_*1…l*_). We calculated the null expectation for the number of samples in each bin (*e*) as simply *e* = *M*/*l*, where M is the number of total measurements made among all cell types and replicates (M = 98 for plant; M = 173 for TCGA data). We then identified the highest bin (*m*) which satisfies o_m_ < e, signifying the transition from abundant noise to high expression. Following the identification of the cutoff region, we calculated the precise background cutoff value by finding the value in the preceding bin that maximized the Spec score.

In both the root data and the TCGA dataset, *l* was set to 10 but results were robust to a range of values of this free parameter. We also set an upper limit on the bin that marked the transition from noise to true expression (*u*), essentially filtering genes that showed a small difference between background and true expression (*u* = 3 for plant data; *u* = 10 for TCGA data).

To select tissue markers, genes were ordered by decreasing Spec score and their cumulative sum was calculated. Markers having the identical Spec score were secondarily ordered by decreasing mean expression in the samples over the background. Top-ranked markers included in the cumulative Spec sum under the threshold (20, unless noted otherwise in the text), and with a minimal tissue Spec score of 0.15, were assigned as tissue markers.

### Index of cell identity calculation

For each cell, a set of ICI scores, one for each tissue (*t*), was calculated as follows.

For each marker gene (*g*), the normalized fragment per million (FPM) expression value (*e*_*g*_) was multiplied by the Spec score for the particular tissue *s*_*g*,*t*_, and the mean of all weighted tissue markers was further weighted by the proportion of markers present (expression > 0):

$$ CI{M}_t=\frac{{\displaystyle {\sum}_g^{n_t}}{e}_g*{s}_{g,t}}{n_t}*\frac{{\displaystyle {\sum}_g^{n_t}} expressed(g)}{n_t} $$

where *n*_*t*_ is the number of markers for tissue *t.*

To determine significance, a population of background ICIs was generated by selecting an equal number of random genes as markers and calculating ICIs for 1,000 permutations. ICIs were considered as significant if they were higher than the top 5% of permutations, unless noted otherwise. ICIs were normalized such that the sum of ICIs for each cell was equal to 1. If more than one identity was significant at the 0.05 threshold level, the cell was considered as chimeric or mixed identity, at the proportions indicated by the ICI scores. If none of the ICIs were significant at the 0.05 level, cell identity was unclassified. All scripts were coded in R 2.15.2, and source code is provided as Additional file 5.

### ICI variability and ICI signal

To calculate ICI variability and ICI signal, the ICI vector for each cell was calculated as detailed above, using a cumulative information threshold that varied between 5 and 100 at increments of 1. Variability at information threshold of *i* was measured as the Euclidean distance between the ICI vectors for information threshold of *i* and *i* + 1. ICI variability for all cells was plotted on the same graph. Signal at information threshold of *i* was the mean maximal ICI value for all cells at that information threshold. Source code is provided as Additional file 5.

### Glioblastoma and mouse embryonic stem cell data analysis

Single cell mRNA-Seq data for mouse embryonic stem cells were obtained from the Gene Expression Omnibus (accession GSE54695) [11], omitting cells with fewer than 1,000 detected genes. A unified scaled dataset for four subtypes of glioblastoma tumors was downloaded from [34] and was used without additional processing to determine Spec scores, as described above. For the glioblastoma tumor data, normalized, scaled single-cell data were obtained from the Gene Expression Omnibus (accession GSE57872) [24]. To obtain FPM values, downloaded data were converted back to absolute values and renormalized by dividing each gene by its mean expression. ICIs were calculated as described above.

### Data access

Data have been submitted to Gene Expression Omnibus, accession numbers GSE46226 (single cell data), GSE64381 (CEL-SEQ profiles) and GSE64253 (QC profiling).

## Additional files

Additional file 1: Figure S1. Variability in marker expressions in single cells. **Figure S2.** Effect of number of markers used on identity specificity. **Figure S3.** The effect of Spec marker selection and the ICI transformation on the affinity of single cells using multidimensional scaling analysis. **Figure S4.** Identity of mixed cells. **Figure S5.** Expression of vascular markers in regenerating stele cells. Additional file 2: Table S1. Datasets used to generate root tissue markers. Additional file 3: Table S2. Spec scores for root tissue markers. Additional file 4: Table S3. Spec scores for glioblastoma markers. Additional file 5:
**R implementation of the algorithm.**

## Abbreviations

CFP: cyan fluorescent protein
DEX: dexamethasone
FPM: fragments per million
GFP: green fluorescent protein
GR: glucocorticoid receptor
ICI: index of cell identity
PI: propidium iodide
QC: quiescent center
TCGA: The Cancer Genome Atlas
