# Supplementary material for: Quantification of cell identity from single-cell gene expression profiles
Source: Genome Biol. 2015 Jan 22;16(1):9. doi: 10.1186/s13059-015-0580-x (PMC4354993; doi:10.1186/s13059-015-0580-x)
Supplement: Additional file 1: Figure S1. — Variability in marker expressions in single cells. Figure S2. Effect of number of markers used on identity specificity. Figure S3. The effect of Spec marker selection and the ICI transformation on the affinity of single cells using multidimensional scaling analysis. Figure S4. Identity of mixed cells. Figure S5. Expression of vascular markers in regenerating stele cells. [file 13059_2015_580_MOESM1_ESM.docx]

**
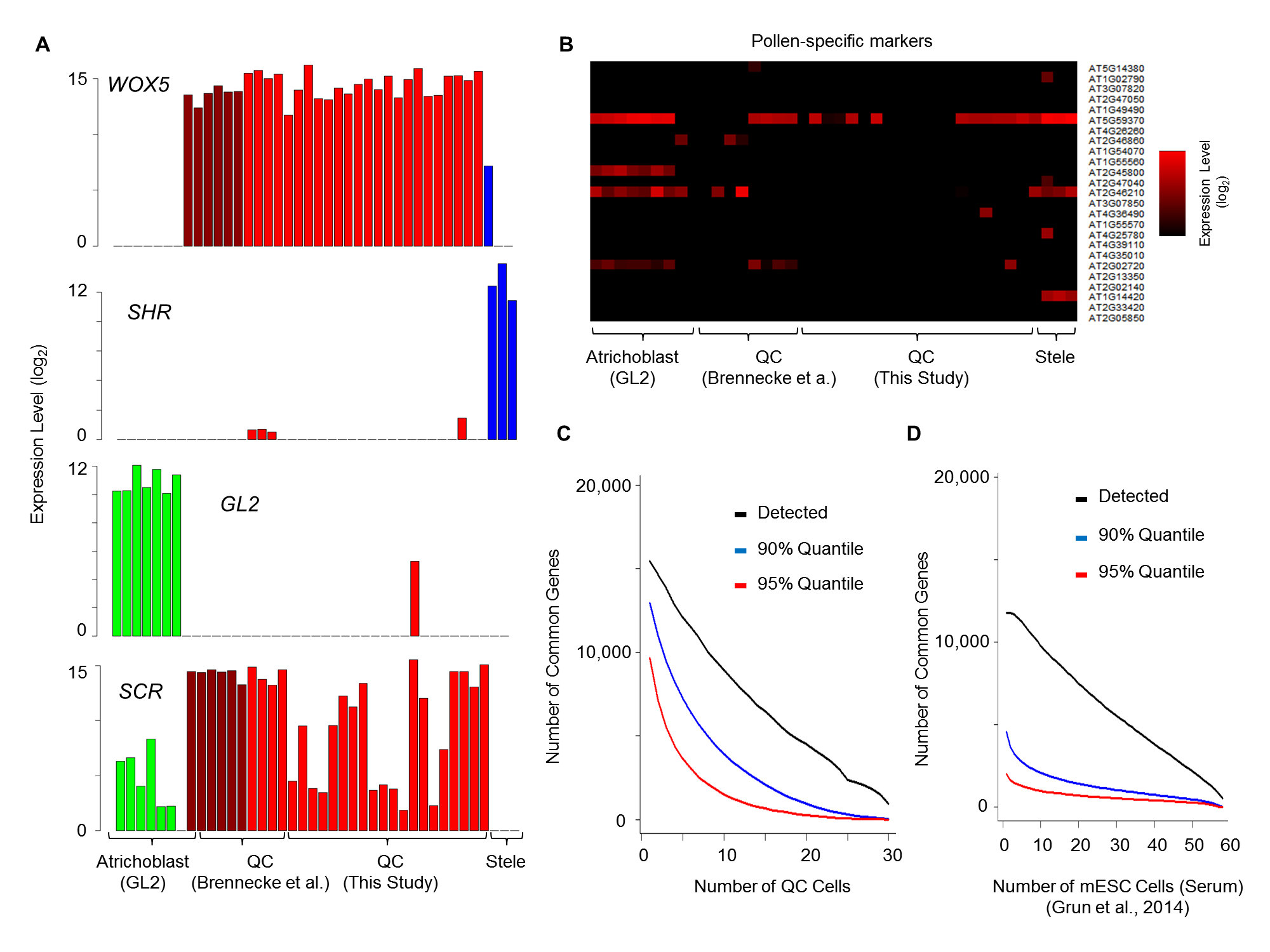
**

**Figure S1. Variability in marker expressions in single cells. A)** Expression level of root cell-type specific markers in single cell samples of root cells, showing detection compared to documented patterns (documented: *WOX5*=QC; *SHR*=stele; *GL2*=atrichoblasts; *SCR*=QC/endodermis. (B) pollen-specific markers in single root cells. **C)** Number of shared expressed genes as a function of the number of QC cells used in the study. For each gene, we calculated the number of QC cells for which the gene was found at the specified expression level (detected or expression in the 90% or 95% quantile). Shown is the total number of genes fitting the different criteria over increasing number of QC cells on the X-axis. **D)** Number of shared expressed genes as a function of the number of cells expressing them among mESC obtained from [11].

**
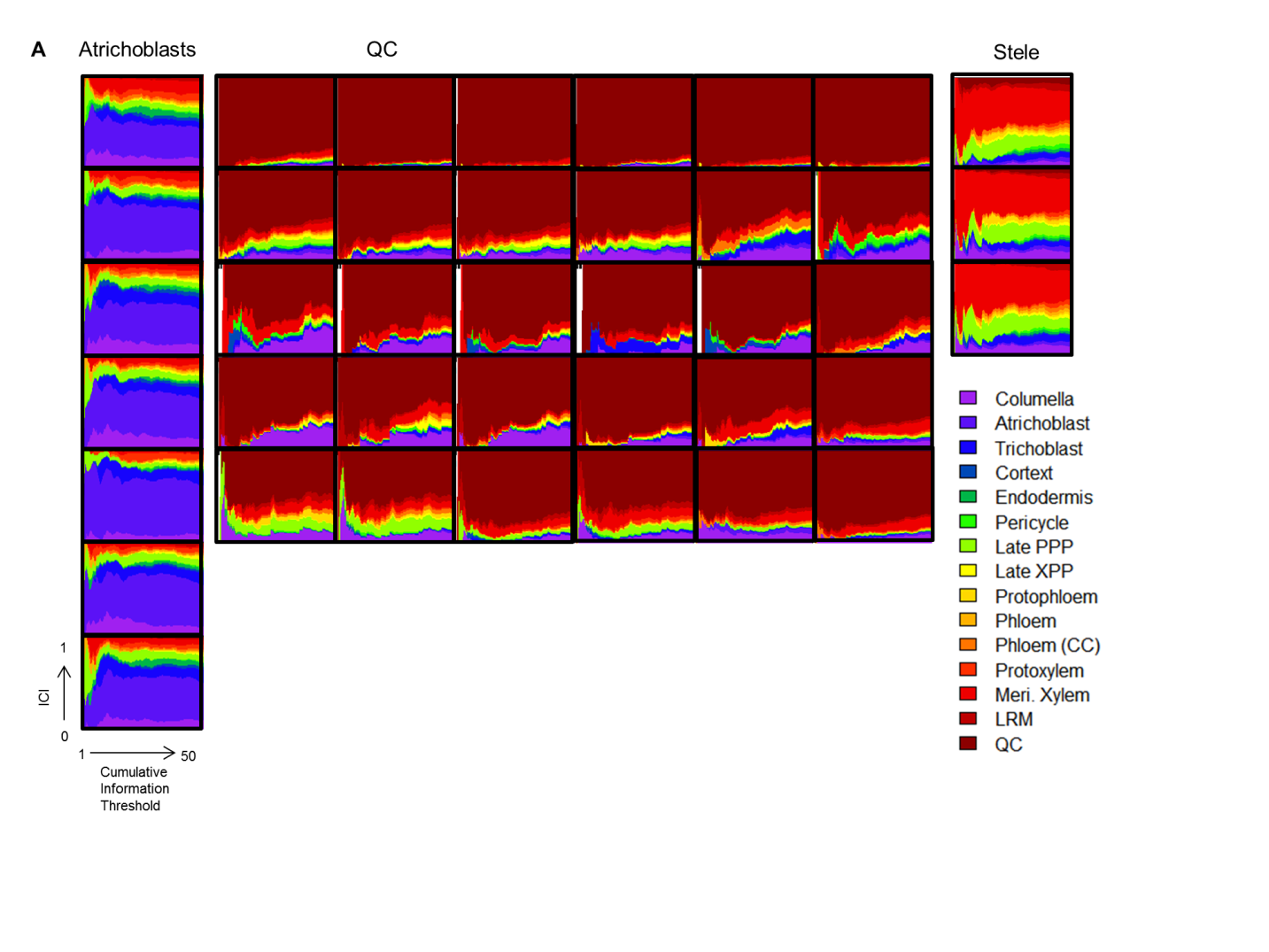
**

**Figure S2. Effect of number of markers used on identity specificity. A)** ICIs of atrichoblasts, QC,and stele cells, as a function of increasing cumulative information threshold, which effectively increases the size of the diagnostic marker set. The identity composition of the calls is on the vertical axis, where different identities are color-coded. The information threshold increases from 1 to 50 along the horizontal axis. Note the high variability (local color fluctuations) in calling primary identity in many cells at a low information threshold. In addition, note the increasing number of false calls (color mixtures), especially in QC and stele, for primary identity as the cumulative information threshold increases.

**
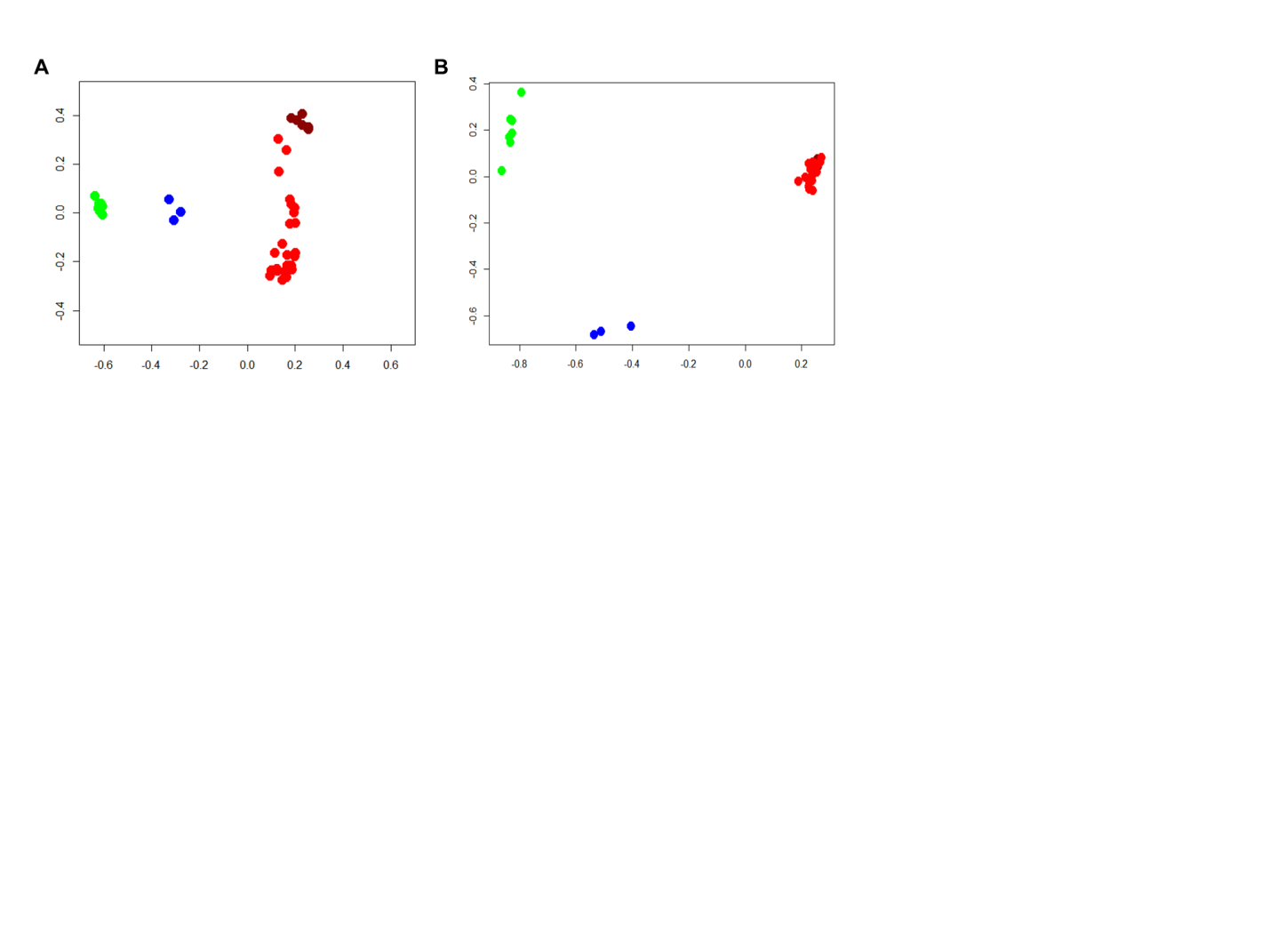
**

**Figure S3. The effect of Spec marker selection and the ICI transformation on the affinity of single cells using an MDS analysis.** **A-B)** MDS using Pearson correlation as a measure of similarity between cells using transcript levels of tissue-specific markers, selected at a cumulative information threshold of 20 (A) and ICI vectors using the same marker set (B). Red – QC, Blue – Stele, Green – Atrichoblasts. Dark red dots specify QC cells from [10].

**
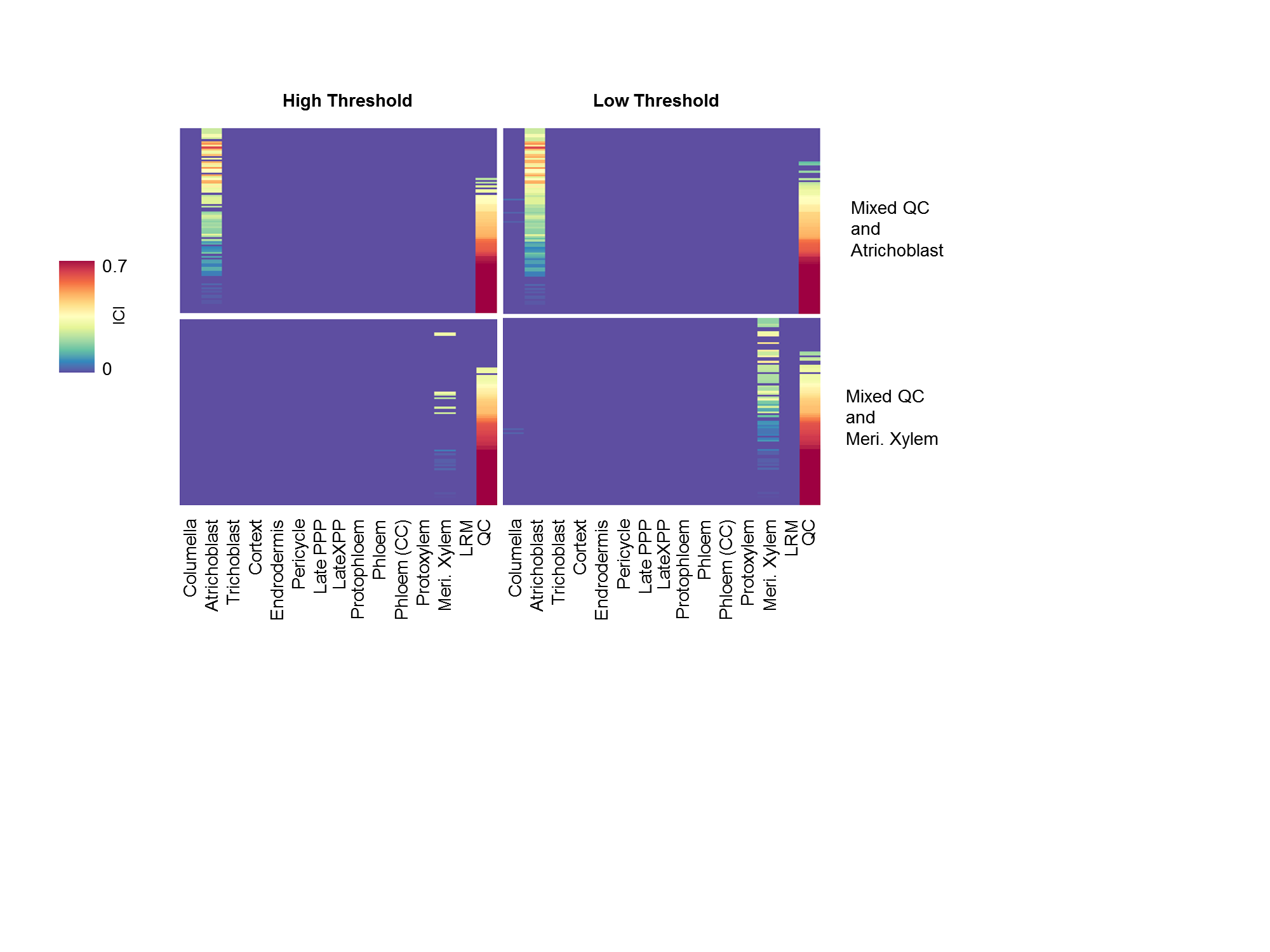
**

**Figure S4. Identity of mixed cells.** ICIs for 100 simulated mixes of QC and atrichoblast cells and 100 QC and stele cells at two significance threshold levels: high (p<0.05), and low (p<0.1). The vertical axis represents different runs of randomly selected reads (see Materials and Methods), where a subset of the original reads are sampled randomly from cells of each type at a 1:1 ratio. Outcomes are ordered on the vertical axis by QC identity score.

**
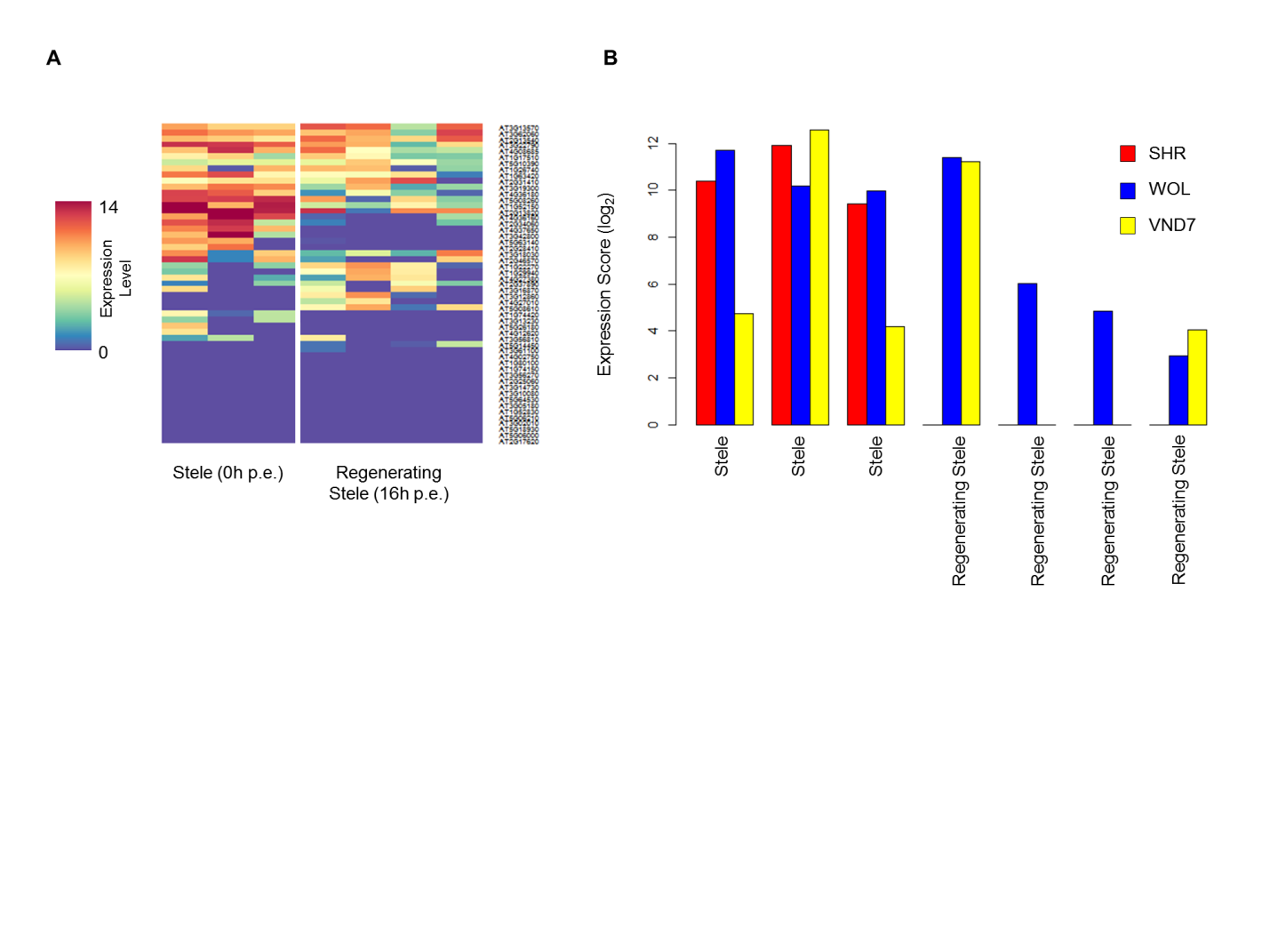
Figure S5. Expression of vascular markers in regenerating stele cells. A)** Expression of meristematic xylem markers in isolated cells from the stele at 0h (immediately after) and 16h post-excision (p.e.). **B)** Expression of the stele markers -- *SHR* and *WOL*, and the xylem marker *VND7* - in stele cells at 0h (left) and 16h (right) p.e.
